# Supplementary material for: ZBP1 promotes LPS-induced cell death and IL-1β release via RHIM-mediated interactions with RIPK1
Source: Nat Commun. 2021 Jan 4;12:86. doi: 10.1038/s41467-020-20357-z (PMC7782486; doi:10.1038/s41467-020-20357-z)
Supplement: Supplementary file 1 — Supplementary Information [file 41467_2020_20357_MOESM1_ESM.pdf]

Supplementary Information

ZBP1 promotes LPS-induced cell death and IL-1β release via RHIM-mediated interactions with RIPK1

Authors: Hayley I. Muendlein, Wilson M. Connolly, Zoie Magri, Irina Smirnova, Vladimir Ilyukha, Avishekh Gautam, Alexei Degterev, Alexander Poltorak

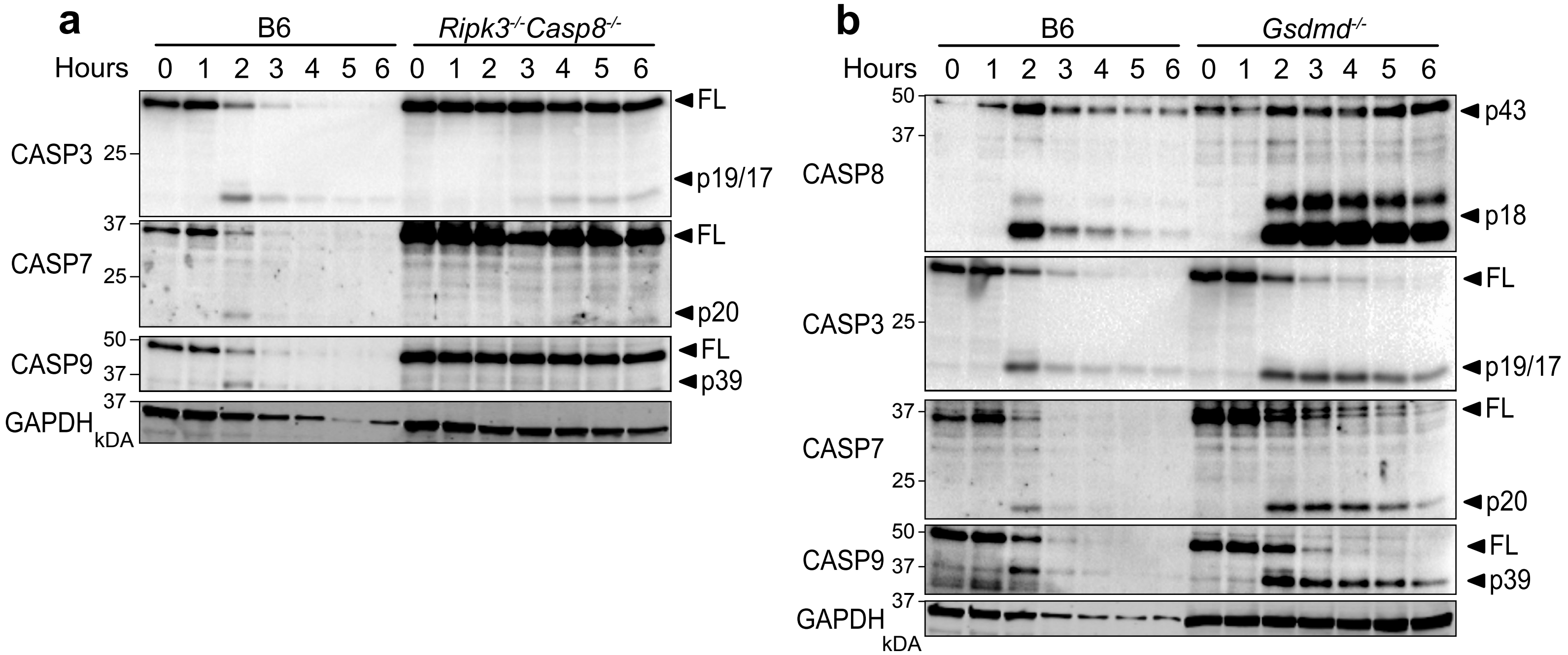

Supplementary Figure 1. (a) Full length and cleaved products of indicated caspases and GSDMD from whole cell lysate of (a) B6 and *Ripk3*$^{-/-}$*Casp8*$^{-/-}$ or (b) B6 and *Gsdmd*$^{-/-}$ BMDMs stimulated with LPS/5z7 for one to six hours. Data from western blots are representative of 3 or more independent experiments. Source data for all experiments are provided as a Source Data file.

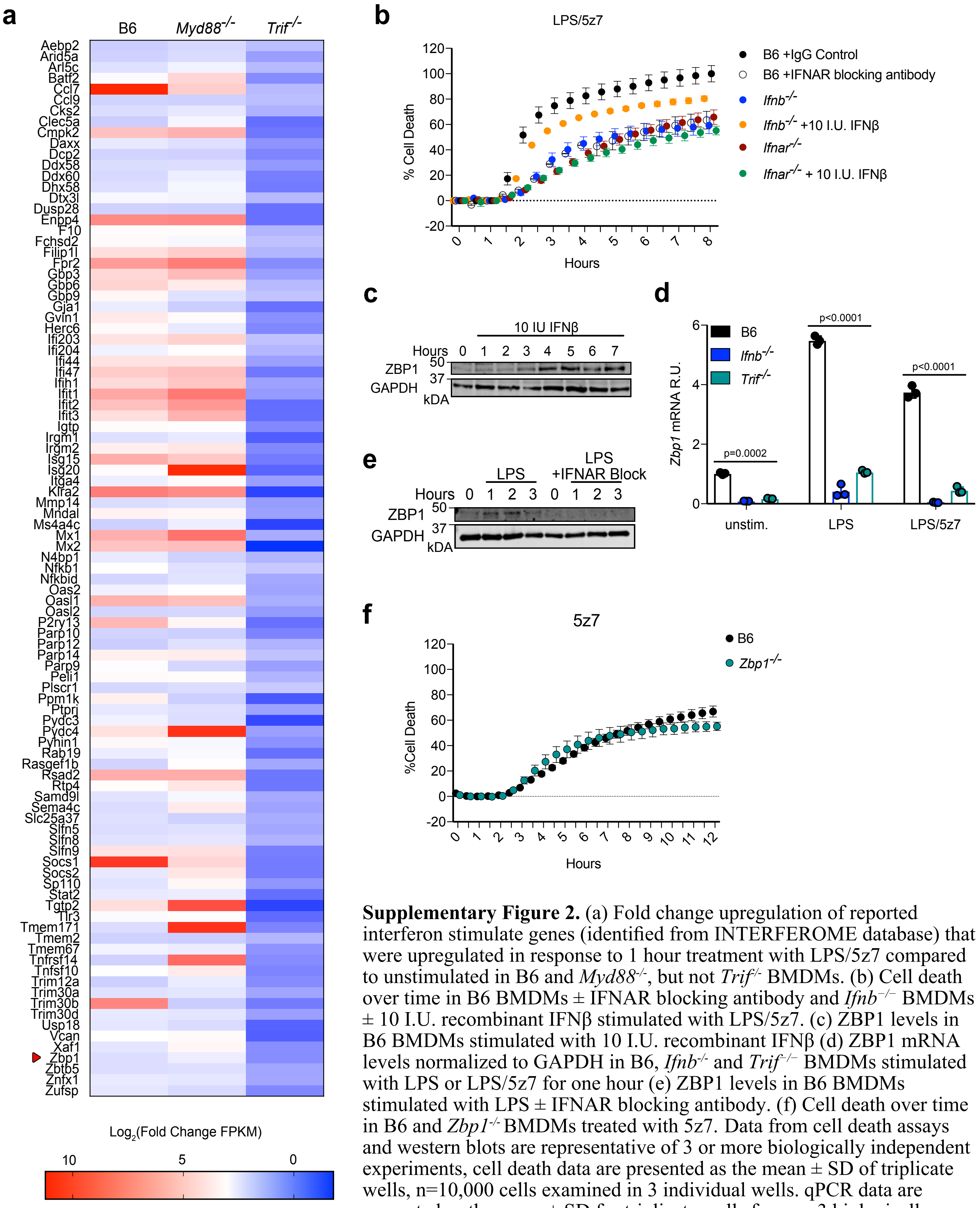

Supplementary Figure 2. (a) Fold change upregulation of reported interferon stimulate genes (identified from INTERFEROME database) that were upregulated in response to 1 hour treatment with LPS/5z7 compared to unstimulated in B6 and *Myd88*$^{-/-}$, but not *Trif*$^{-/-}$ BMDMs. (b) Cell death over time in B6 BMDMs ± IFNAR blocking antibody and *Ifnb*$^{-/-}$ BMDMs ± 10 I.U. recombinant IFNβ stimulated with LPS/5z7. (c) ZBP1 levels in B6 BMDMs stimulated with 10 I.U. recombinant IFNβ (d) ZBP1 mRNA levels normalized to GAPDH in B6, *Ifnb*$^{-/-}$ and *Trif*$^{-/-}$ BMDMs stimulated with LPS or LPS/5z7 for one hour (e) ZBP1 levels in B6 BMDMs stimulated with LPS ± IFNAR blocking antibody. (f) Cell death over time in B6 and *Zbp1*$^{-/-}$ BMDMs treated with 5z7. Data from cell death assays and western blots are representative of 3 or more biologically independent experiments, cell death data are presented as the mean ± SD of triplicate wells, n=10,000 cells examined in 3 individual wells. qPCR data are presented as the mean ± SD for triplicate wells from n=3 biologically independent experiments. Two-way Analysis of variance (ANOVA) was used for comparison between groups. Source data for all experiments are provided as a Source Data file.

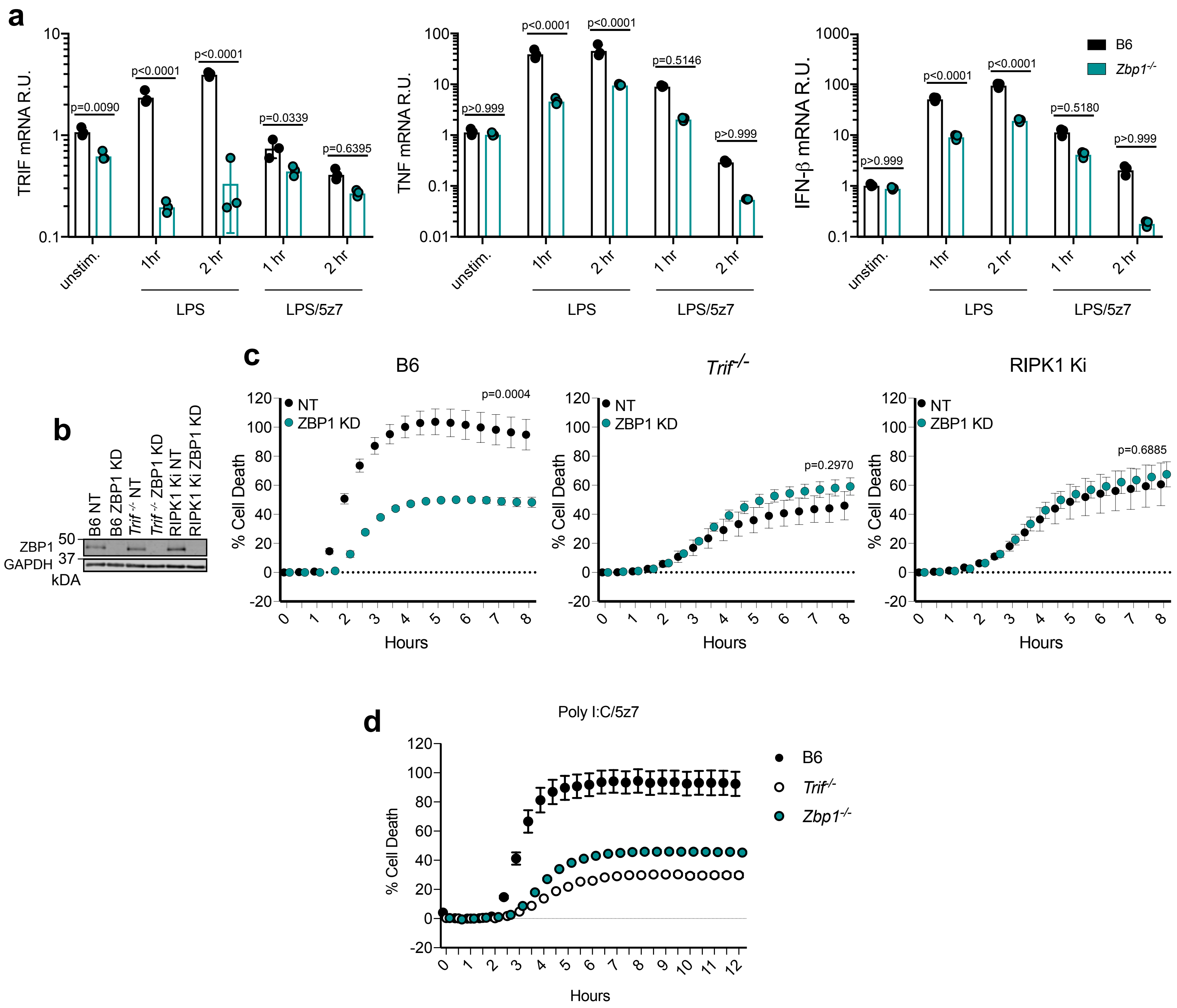

Supplementary Figure 3. (a) TRIF, TNF and IFNβ mRNA levels normalized to GAPDH in B6, and *Zbp1*$^{-/-}$ BMDMs stimulated with LPS or LPS/5z7 for one or 2 hours. (b) Confirmation of ZBP1 knock down (KD) in B6, *Trif*$^{-/-}$ and RIPK1 Ki BMDMs. (c) Cell death overtime as measured by propidium iodide (PI) incorporation in B6, *Trif*$^{-/-}$ and RIPK1 Ki BMDMs ± ZBP1 KD stimulated with LPS/5z7. (d) Cell death overtime as measured by PI incorporation in B6, *Trif*$^{-/-}$ and *Zbp1*$^{-/-}$ BMDMs stimulated with Poly I:C/5z7. Data from cell death assays and western blots are representative of 3 or more biologically independent experiments, cell death data are presented as the mean ± SD of triplicate wells, n=10,000 cells examined in 3 individual wells. qPCR data are presented as the mean ± SD for triplicate wells from n=3 biologically independent experiments. Two-way Analysis of variance (ANOVA) was used for comparison between groups. Source data for all experiments are provided as a Source Data file.

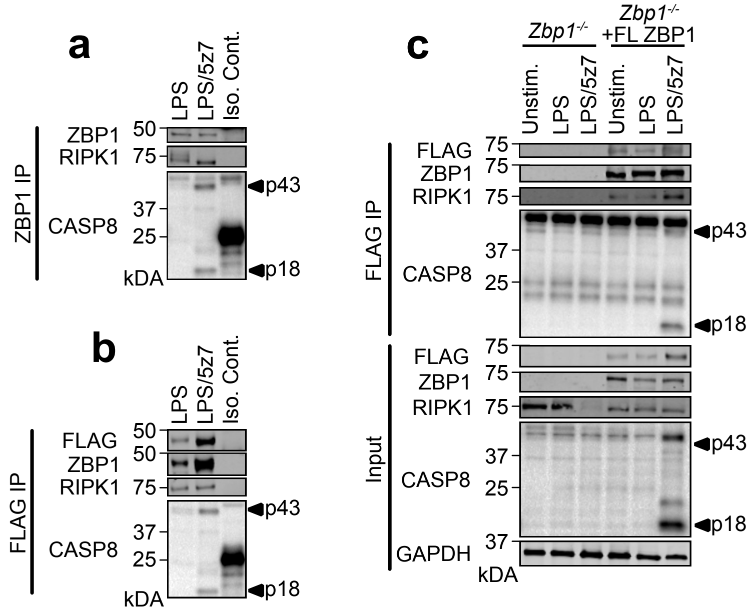

Supplementary Figure 4. (a) ZBP1-specific immunoprecipitation in B6 BMDMs stimulated as indicated and run with isotype control. (b) FLAG-specific immunoprecipitation in B6 BMDMs stimulated as indicated and run with isotype control. (c) FLAG-specific immunoprecipitation in *Zbp1*$^{-/-}$ BMDMs ± reconstitution with FLAG-tagged Full length ZBP1 (+FL ZBP1) stimulated as indicated. Data from western blots are representative of 3 or more biologically independent experiments. Source data for all experiments are provided as a Source Data file.

Target	Forward Primer	Reverse Primer
IFNb	CAGCTCCAAGAAAGGACGAAC	GGCAGTGTAACTCTTCTGCAT
ACTB	CACTGTCGAGTCGCGTCCA	GACCCATTCCCACCATCACA
TRIF	TACCAGCTCAAGACCCCTACA	GTCCCTTTCCAAGGCACCTA
TNF	CTGTAGCCCACGTCGTAGC	TTGAGATCCATGCCGTTG
ZBP1	AGCCATTCTTGCCTGTGGATT	ACACCTGCAGGATCTTTTGC

Supplementary Table 1. Forward and reverse primers used for quantitative PCR analyses.
