# Supplementary material for: ZBP1 promotes LPS-induced cell death and IL-1β release via RHIM-mediated interactions with RIPK1
Source: Nat Commun. 2021 Jan 4;12:86. doi: 10.1038/s41467-020-20357-z (PMC7782486; doi:10.1038/s41467-020-20357-z)
Supplement: Supplementary file 3 — Reporting Summary [file 41467_2020_20357_MOESM3_ESM.pdf]

nature research

Corresponding author(s): Alexander Poltorak

Last updated by author(s): Nov 10, 2020

Reporting Summary

Nature Research wishes to improve the reproducibility of the work that we publish. This form provides structure for consistency and transparency in reporting. For further information on Nature Research policies, see our Editorial Policies and the Editorial Policy Checklist.

Statistics

For all statistical analyses, confirm that the following items are present in the figure legend, table legend, main text, or Methods section.

n/a	Confirmed	
☐	☒	The exact sample size (*n*) for each experimental group/condition, given as a discrete number and unit of measurement
☐	☒	A statement on whether measurements were taken from distinct samples or whether the same sample was measured repeatedly
☐	☒	The statistical test(s) used AND whether they are one- or two-sided<br>*Only common tests should be described solely by name; describe more complex techniques in the Methods section.*
☒	☐	A description of all covariates tested
☒	☐	A description of any assumptions or corrections, such as tests of normality and adjustment for multiple comparisons
☐	☒	A full description of the statistical parameters including central tendency (e.g. means) or other basic estimates (e.g. regression coefficient) AND variation (e.g. standard deviation) or associated estimates of uncertainty (e.g. confidence intervals)
☒	☐	For null hypothesis testing, the test statistic (e.g. *F*, *t*, *r*) with confidence intervals, effect sizes, degrees of freedom and *P* value noted<br>*Give P values as exact values whenever suitable.*
☒	☐	For Bayesian analysis, information on the choice of priors and Markov chain Monte Carlo settings
☒	☐	For hierarchical and complex designs, identification of the appropriate level for tests and full reporting of outcomes
☒	☐	Estimates of effect sizes (e.g. Cohen's *d*, Pearson's *r*), indicating how they were calculated

Our web collection on statistics for biologists contains articles on many of the points above.

Software and code

Policy information about availability of computer code

Data collection	BioTek Gen5 Imaging Software 3.08, Li-COR ImageStudio Western Blot Software
Data analysis	BioTek Gen5 Imaging Software 3.08, Li-COR ImageStudio Western Blot Software, TopHat2 and Cufflinks Software

For manuscripts utilizing custom algorithms or software that are central to the research but not yet described in published literature, software must be made available to editors and reviewers. We strongly encourage code deposition in a community repository (e.g. GitHub). See the Nature Research guidelines for submitting code & software for further information.

Data

Policy information about availability of data

All manuscripts must include a data availability statement. This statement should provide the following information, where applicable:

- Accession codes, unique identifiers, or web links for publicly available datasets
- A list of figures that have associated raw data
- A description of any restrictions on data availability

The RNA-Sequencing data that support the findings of this study have been deposited in the GenBank Gene Expression Omnibus (GEO) Database with the primary accession code GSE83885.
Reported interferon stimulated genes within our dataset were identified using the INTERFEROME database (http://www.interferome.org/interferome/site/showCitation.jspx)

Field-specific reporting

Please select the one below that is the best fit for your research. If you are not sure, read the appropriate sections before making your selection.

[x] Life sciences [ ] Behavioural & social sciences [ ] Ecological, evolutionary & environmental sciences

For a reference copy of the document with all sections, see nature.com/documents/nr-reporting-summary-flat.pdf

Life sciences study design

All studies must disclose on these points even when the disclosure is negative.

Sample size	For each finding presented, experiments were conducted on 100 thousand to 10 million bone marrow derived macrophages per condition depending on specific output required in biologic and technical triplicate.
Data exclusions	No Data were excluded
Replication	For every finding presented, experiments were conducted in at least biological and technical triplicate to ensure reproducibility between biological samples and experimental runs.
Randomization	Mice used for isolation of bone marrow derived macrophages (males and females 6-12 weeks) were chosen randomly. When cells were plated for various experiments, plating schemes were randomized to avoid plating or imaging biases.
Blinding	For our study, blinding is not necessary since our readouts such as % of cell death, protein and mRNA levels and extent of protein cleavage are not largely subjective.

Reporting for specific materials, systems and methods

We require information from authors about some types of materials, experimental systems and methods used in many studies. Here, indicate whether each material, system or method listed is relevant to your study. If you are not sure if a list item applies to your research, read the appropriate section before selecting a response.

Materials & experimental systems

n/a	Involved in the study	
[ ]	[x]	Antibodies
[ ]	[x]	Eukaryotic cell lines
[x]	[ ]	Palaeontology and archaeology
[ ]	[x]	Animals and other organisms
[x]	[ ]	Human research participants
[x]	[ ]	Clinical data
[x]	[ ]	Dual use research of concern

Methods

n/a	Involved in the study	
[x]	[ ]	ChIP-seq
[x]	[ ]	Flow cytometry
[x]	[ ]	MRI-based neuroimaging

Antibodies

Antibodies used	Primary antibodies against Casp8 (8592), Casp3 (9665), Casp7 (9492), Casp9 (9508), Casp1 (24232), Casp11 (14340), RIPK1 (3493), pRIPK1 (Ser166) (31122), pRIPK1 (Ser321) (38662), IL-1β (12242), FLAG (8146) and GAPDH (2118) were purchased from Cell Signaling Technologies. ZBP1 antibody (AG-20B-0010-C100) was purchased from Adipogen. GSDMD antibody (ab209845) was purchased from Abcam. FADD antibody (05-486) was purchased from Millipore Sigma. Secondary antibodies, anti-rabbit IgG (H+L) (DyLightTM 800 4X PEG Conjugate) (5151), anti-mouse IgG (H+L) (DyLightTM 800 4X PEG Conjugate) (5257) and HRP-linked anti-rat IgG antibody (7077) were purchased from Cell Signaling Technologies. Anti-ASC antibody (Cell Signaling Technologies, 67824). Alexa Fluor 488-conjugated-Goat-anti-Rabbit IgG (Invitrogen A-11034). Mouse TNF neutralizing antibody was purchased from Cell Signaling Technology (11969S).
Validation	All primary antibodies used were validated for their use in western blot and/or immunoprecipiation procedures as indicated according to manufacturer's website. All primary antibodies used were validated for reactivity in mouse cells according to the manufacturer's website. Antibodies were also confirmed using positive and negative controls within our lab whenever possible.

Eukaryotic cell lines

Policy information about cell lines

Cell line source(s)	Immortalized Rip3Casp8−/−, ZBP1−/−, and B6 bone marrow derived macrophages were generated from primary bone marrow derived macrophages from indicated mouse strains and donated by Dr. K. Fitzgerald. L929, 293T cell lines were purchased from ATCC.

Authentication	All immortalized cell lines were compared to primary bone marrow derived macrophages from mice of the same genotype to ensure that phenotypes were consistent for all findings presented after the immortalization process.
Mycoplasma contamination	cell lines were not tested for mycoplasma contamination.
Commonly misidentified lines (See ICLAC register)	N/A

Animals and other organisms

Policy information about studies involving animals; ARRIVE guidelines recommended for reporting animal research

Laboratory animals	C57BL/6 (B6), Ticam1−/− (Trif−/−, C57BL/6J-Ticam1Lps2/J), Cd14−/− (B6.129S4-Cd14tm Frm/J), Myd88−/− (B6.129P2(SJL)-Myd88tm1.1Defr/J), Tnfr−/− (C57BL/6-Tnfrsf1atm1lmx/J) and Nlrp3-/- (B6.129S6-Nlrp3tm1Bhk/J) mice were obtained from The Jackson Laboratory. Ifnb−/− (C57BL/6 background) were a gift from Dr. S. Vogel. Mice were housed according to protocols approved by the Tufts University Medical School Animal Care and Use Committees. Femurs from Ticam2-/- (Tram−/−, C57BL/6 background) mice were generously donated by Dr. L. Li. Femurs from Ripk3-/- (C57BL/6 background) and Gsdmd −/− (C57BL/6 background) mice were donated by Dr. K. Fitzgerald, and were generated by Dr. V. Dixit. Ripk1K45A/K45A (RIPK1 Ki, C57BL/6 background) mice were provided by Dr. A. Degterev. Femurs from Ripk3-/-Casp8−/− (C57BL/6 background) mice were donated by Dr. K. Fitzgerald, and were originally generated by Dr. D. Green. Femurs from Zbp1−/− mice (C57BL/6 background) were generously donated by Dr. S. Balachandran. Bones from Casp3-/-Casp7-/- (C57BL/6 background) were generated and provided by Dr. A. Rongvaux. Bone marrow was isolated from the long bones of 6-12 week old male and female mice to generate bone marrow derived macrophages used for experiments.
Wild animals	N/A
Field-collected samples	N/A
Ethics oversight	Mice were housed according to protocols approved by the Tufts University Medical School Animal Care and Use Committees.

Note that full information on the approval of the study protocol must also be provided in the manuscript.
